# Reduction in systemic muscle stress markers after exercise-induced muscle damage following concurrent training and supplementation with specific collagen peptides – a randomized controlled trial

**DOI:** 10.3389/fnut.2024.1384112

**Published:** 2024-03-25

**Authors:** Kevin Bischof, Savvas Stafilidis, Larissa Bundschuh, Steffen Oesser, Arnold Baca, Daniel König

**Affiliations:** ^1^Centre for Sports Science and University Sports, Department of Sports Science, Section for Nutrition, Exercise and Health, University of Vienna, Vienna, Austria; ^2^Vienna Doctoral School of Pharmaceutical, Nutritional and Sport Sciences, University of Vienna, Vienna, Austria; ^3^Centre for Sports Science and University Sports, Department for Biomechanics, Kinesiology and Computer Science in Sport, University of Vienna, Vienna, Austria; ^4^CRI, Collagen Research Institute, Kiel, Germany; ^5^Faculty of Life Sciences, Department for Nutrition, Section for Nutrition, Exercise and Health, University of Vienna, Vienna, Austria

**Keywords:** collagen peptides, muscle damage, concurrent training, recovery, regeneration, creatine kinase, repeated bout effect

## Abstract

**Introduction:**

Collagen peptide supplementation in conjunction with exercise has been shown to improve structural and functional adaptations of both muscles and the extracellular matrix. This study aimed to explore whether specific collagen peptide (SCP) supplementation combined with a concurrent training intervention can improve muscular stress after exercise-induced muscle damage, verified by reliable blood markers.

**Methods:**

55 sedentary to moderately active males participating in a concurrent training (CT) intervention (3x/week) for 12 weeks were administered either 15 g of SCP or placebo (PLA) daily. Before (T1) and after the intervention (T2), 150 muscle-damaging drop jumps were performed. Blood samples were collected to measure creatine kinase (CK), lactate dehydrogenase (LDH), myoglobin (MYO) and high-sensitivity C-reactive protein (hsCRP) before, after, and at 2 h, 24 h and 48 h post exercise.

**Results:**

A combination of concurrent training and SCP administration showed statistically significant interaction effects, implying a lower increase in the area under the curve (AUC) of MYO (*p* = 0.004, ηp^2^ = 0.184), CK (*p* = 0.01, ηp^2^ = 0.145) and LDH (*p* = 0.016, ηp^2^ = 0.133) in the SCP group. On closer examination, the absolute mean differences (ΔAUCs) showed statistical significance in MYO (*p* = 0.017, *d* = 0.771), CK (*p* = 0.039, *d* = 0.633) and LDH (*p* = 0.016, *d* = 0.764) by SCP supplementation.

**Conclusion:**

In conclusion, 12 weeks of 15 g SCP supplementation combined with CT intervention reduced acute markers of exercise-induced muscle damage and improved post-exercise regenerative capacity, as evidenced by the altered post-exercise time course. The current findings indicate that SCP supplementation had a positive effect on the early phase of muscular recovery by either improving the structural integrity of the muscle and extracellular matrix during the training period or by accelerating membrane and cytoskeletal protein repair.

**Clinical trial registration:**

https://www.clinicaltrials.gov/study/NCT05220371?cond=NCT05220371&rank=1, NCT05220371.

## 1 Introduction

Tissues responsible for proper movement execution (e.g., muscles, tendons) underlie a perpetual process of adaptation in response to specific environmental stimuli. In particular, unaccustomed eccentric exercise is known to induce high mechanical stress, leading to myofibril damage and extracellular matrix (ECM) disruption (1). Many studies have focused on nutritional interventions aimed at improving recovery from locomotor impairments, which are often observed as reductions in functional movement abilities (2–8). In this context, interest in collagen peptide (CP) administration has increased in recent years since regular CP intake has been shown to possibly alleviate joint discomfort, enhance ankle and knee functionality and improve recovery from Achilles tendinopathy, therefore acting as a potential and safe nutritional supplement in sports related injuries (9). Moreover, several short-term studies have reported that CP ingestion is superior to placebo after exercise-induced muscle damage (10–13). Faster restoration of biomechanical parameters such as improved countermovement jump height at 24 h (12) and 48 h (10), improved post-exercise muscle strength at 48 h, and reduced muscle soreness immediately after a muscle-damaging exercise bout (13) have been demonstrated following CP intake in conjunction with exercise. A proteomic analysis illustrated a significantly higher degree of upregulated proteins associated with skeletal muscle fibers, mainly contraction-related changes and structural adaptations to training. This suggests greater changes in collagen-specific proteins after 12 weeks of CP supplementation combined with resistance training (RT) (14). A recent study investigating gene expression patterns following strenuous bouts of knee extensions prior to a single dose of CP showed significant upregulation of the PI3K/Akt and MAPK pathways, both known to be essential for (myofibrillar) protein synthesis (15). The PI3K/Akt pathway may also play a role in connective tissue protein synthesis by mediating TGF-β2 signaling and inducing mRNA expression of COL1A1 & COL1A2 (collagen type 1 genes) in human pigment cells (16). Whether CP supplementation also stimulates connective tissue protein synthesis rates *in vivo* is currently unknown (17), but seems plausible, as increased collagen concentration was measured in human *in vitro*-engineered ligament after a single dose of 15 g CP (18).

Recovery from severe lengthening contractions (e.g., squats or drop jumps) also causes biomarkers in serum blood to peak within the following days. Proteins and metabolic-related enzymes such as creatine kinase (CK), lactate dehydrogenase (LDH), myoglobin (MYO), alanine transaminase (ALT), c-reactive protein (CRP) and interleukin-6 (IL-6) have been considered metabolic indicators of muscle damage and concomitant inflammation, both of which occur after strenuous, mainly eccentric exercise (19). However, there are only two studies without exercise intervention that examined recovery-related blood markers after CP supplementation. Neither found differences between CP and placebo after nine and 42 days of supplementation (10, 11). Therefore, the importance of regular, long-term, and at least moderately intense physical activity in combination with CP ingestion may be of interest for proteomic outcomes related to recovery. Furthermore, the CP administered in this study have only been utilized in two long-term investigations without examining blood related recovery parameters so far (20, 21). Thus, the impact of these particular CPs is also noteworthy.

Based on this assumption, the present study aimed to investigate whether 12 weeks of CP supplementation together with a concurrent training intervention would significantly influence exercise-induced muscular stress. In addition, recovery should be confirmed by specific blood parameters. This could indicate either a reduced acute stress response due to improved functional or structural integrity of the muscles. Or, on the other hand, an optimized muscular regenerative capacity that alleviates both myofibrillar damage and inflammation after exercise-induced muscle damage, or both. Therefore, we hypothesized that the increase of biomarkers in blood after a second bout of muscle-damaging exercise would be lower in subjects taking specific collagen peptides (SCP).

## 2 Methods

### 2.1 Study design

The study was a randomized, double-blind, placebo-controlled trial conducted at the University of Vienna, Austria. The study was approved by the Ethics Committee of the University of Vienna (reference no. 00765) and registered at ClinicalTrials.gov (ID: NCT05220371). 75 healthy male participants aged 18–40 years who had not exercised more than 3 hours per week in the preceding months were randomized to the treatment or placebo group after signing a written informed consent. The IPAQ questionnaire quantified individual physical activity levels. Exclusion criteria were a body mass index (BMI) below 18.5 kg/m^2^ or above 25 kg/m^2^, unstable weight or dietary behavior, physical complaints related to physical activity, and intake of protein supplements within the 6 months before the start of the study. In addition, subjects with renal, cardiovascular or metabolic diseases were excluded in accordance with American College of Sports Medicine (ACSM) guidelines (22).

**Figure 1:**
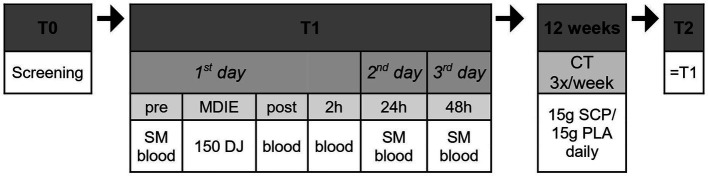
Study design in chronological order. Blood, blood collection; MDIE, muscle damage inducing exercise; DJ, drop jump; CT, concurrent training; SM, standardized meal.

The study design (Figure 1) comprised pre-tests (T1) before the training intervention, 12 weeks with daily intake of SCP or PLA with concurrent training (3x/week), and study completion with subsequent post-tests (T2) corresponding to the pre-tests. Subjects were screened 2 weeks before T1. Using a research randomizer^1^, participants were assigned to the SCP or PLA group after randomization. At baseline, study participants were given a standardized meal (SM) after overnight fasting (7 to 9 a.m.). 1 hour after the SM (“pre”), the first blood samples were collected, followed by the performance of 150 drop jumps. Immediately and 2 hours later, blood samples were collected again (“post,” “2 h”). On the second and third days, subjects arrived at the same time as on day 1, received their SM and waited 1 hour until blood was drawn two more times (“24 h,” “48 h”). Subjects were required to refrain from physically demanding or unaccustomed exercise and alcohol consumption for 48 h before the trials. Only water was allowed during the test procedures except for the SM.

### 2.2 Blood samples

Each blood sample was collected venously via venipuncture and filled into 10 mL serum vacutainers. These were centrifuged at 2500 rpm for 15 min, and the resulting supernatant was pipetted into 2 mL Eppendorf tubes and immediately stored in a refrigerator at -32°C. For analysis purposes, the frozen tubes were transported to a local laboratory and analyzed separately. The Beckman Coulter AU5822 (23) was used for detecting creatine kinase (CK), lactate dehydrogenase (LDH) and myoglobin (MYO). High-sensitivity C-reactive protein (hsCRP) was detected “pre” & “24 h” by immunoturbidimetry (Alinity c CRP Vario Reagent Kit, Abbott Laboratories) with a coefficient of variance (CV) of 12.5%. The “24 h” measurement was chosen due to the high likelihood of CRP levels peaking 1 day after intense exercise (24, 25). As some participants were unable to provide blood at the given times, they were excluded from the data analysis.

### 2.3 Muscle damage-inducing exercise (MDIE)

The MDIE employed consisted of 150 drop jumps from a 60 cm box. Six sets of 25 jumps were prescribed, with a two-minute rest after each set. Participants had to perform the drop jumps slowly and achieve a knee angle of at least 90° to produce sufficient muscle damage, which has been documented elsewhere (26).

### 2.4 Supplementation

15 g of either a placebo (Silicea) or the test product (a mixture of specific collagen peptides [10 g PeptENDURE® + 5 g TENDOFORTE®], Gelita AG, Eberbach, Germany, Table 1) were administered orally daily for 12 weeks in powder form. Both products were mixed with 500 mL of water and drunk. One half of the SCP and PLA packs had to be taken 1 hour before and the second half instantly after the training session. This administration schedule would result in high blood levels of collagen peptides during and after exercise (27–29). On non-training days, the participants consumed 15 g as a whole at the same time as on exercise days.

**Table 1:** Amino acids of the specific collagen peptides.

Amino acid	Weight (%)
Glycine	22.1
Proline	12.3
Hydroxyproline	11.3
Glutamic acid	10.1
Alanine	8.5
Arginine	7.8
Aspartic acid	5.8
Lysine	3.8
Serine	3.2
Leucine	2.7
Valine	2.4
Phenylalanine	2.1
Threonine	1.8
Hydroxylysine	1.7
Isoleucine	1.3
Histidine	1.2
Tyrosine	0.9
Methionine	0.9

### 2.5 Exercise protocol

A concurrent training (CT) intervention was conducted three times weekly for 12 weeks. The first half of the exercise program consisted of lower body weight exercises (squats, lunges and calf raises in chronological order). Starting with three sets of 20 repetitions for each exercise, five repetitions were added after 4 and 8 weeks. For the last 30 min of the CT, subjects ran at a heart rate determined individually according to the Karvonen formula (30) (training intensity factor between 0.7–0.8; HRmax = 220 – age). Pulse watches were provided for each training session to maintain the pace. After 4 and 8 weeks, the individual training intensity factor was increased by 5%. Recovery was ensured by a rest day between two training sessions. Training sessions were conducted outdoors every Monday, Wednesday and Friday at 7 a.m. and 6 p.m. under the supervision of sport scientists at the Institute of Sport Science in Vienna. Participants were encouraged to exercise regularly, either in the morning or afternoon session. Subjects completing fewer than 26 exercise sessions were eliminated from the study.

### 2.6 Standardized meal (SM)

The SM (oats and an oat drink) was provided at T1 & T2 on every test day, which participants consumed 2 hours before blood collection. The SM contained 1 g of carbohydrate per kg of body weight. In addition, participants were required to ingest 15 g of PLA or SCP with their meal at T2.

### 2.7 Dietary intake

Using household measurements, participants were required to quantify all the beverages and foods they consumed on two weekdays and one weekend day in weeks 1 and 12. Nut.s (nutritional.software, dato Denkwerkzeuge, Vienna) analyzed the amount of total energy intake, carbohydrates, fats and proteins.

### 2.8 Statistical analysis

IBM SPSS Statistics 23 (IBM SPSS Statistics for Windows, Armonk, NY: IBM Corp.) was used to perform all statistical analyses. Figures were generated by GraphPad Prism 9. The α-level was set to 5%. Blood marker analysis was based on the data listed in Table 2. These data were used to calculate the area under the curve (AUC) with the linear trapezoidal rule for each blood biomarker (31) and the deltas of these AUCs. Normal distribution was demonstrated with the Kolmogorov–Smirnov test. Dependent t-tests elaborated changes over time. Variance of homogeneity was tested with Levene’s test as a prerequisite for the independent *t*-test. A mixed design ANOVA (generalized linear model with repeated measures) was used for interaction effects, with group (SCP, PLA) as a between-subjects factor and study time (T1, T2) as a within-subjects factor. Partial eta-square (ηp^2^) was given as effect size (small effect: ηp^2^ > 0.01, medium effect: ηp^2^ > 0.06, large effect: ηp^2^ > 0.14). The effect size in pairwise t-tests was expressed as Cohen’s d (small effect: >0.2, medium effect: >0.5, large effect: >0.8) (32). In the case of a significant Mann–Whitney U test, the effect size was also reported as Cohen’s d/ηp^2^ [Wolfgang Lenhard & Alexandra (33)]. All tests were two-sided. Due to increased variability in the data sets, outliers were eliminated if they were above the 3rd standard deviation.

**Table 2:** Concentrations/activity of recovery-related blood biomarkers represented as mean ± SD.

Group	Time									
	T1					T2				
	pre	post	2 h	24 h	48 h	pre	post	2 h	24 h	48 h
CK (U/L)										
PLA (*n* = 23)	135 ± 64	159 ± 69	182 ± 66	346 ± 197	246 ± 123	142 ± 64	161 ± 66	171 ± 64	214 ± 108	207 ± 140
CP (*n* = 22)	136 ± 65	174 ± 77	204 ± 88	422 ± 225	506 ± 782	125 ± 66	146 ± 74	156 ± 71	192 ± 133	162 ± 103
LDH (U/L)										
PLA (*n* = 21)	165 ± 21	181 ± 24	180 ± 24	174 ± 31	175 ± 35	161 ± 30	176 ± 29	176 ± 29	173 ± 27	178 ± 31
CP (*n* = 22)	181 ± 20	208 ± 21	204 ± 22	195 ± 24	190 ± 22	175 ± 24	185 ± 24	185 ± 24	182 ± 23	188 ± 26
MYO (ng/ml)										
PLA (*n* = 23)	51 ± 8	89 ± 43	141 ± 108	55 ± 21	49 ± 14	46 ± 9	59 ± 32	77 ± 45	45 ± 13	47 ± 16
CP (*n* = 21)	48 ± 13	94 ± 50	165 ± 93	62 ± 29	96 ± 120	40 ± 13	46 ± 13	51 ± 14	36 ± 12	35 ± 10
hsCRP (mg/l)										
PLA (*n* = 21)	0.67 ± 0.4	-	-	1.04 ± 1	-	0.61 ± 0.3	-	-	0.55 ± 0.2	-
CP (*n* = 19)	1.46 ± 1.4	-	-	2.2 ± 1.8	-	1.21 ± 0.9	-	-	1.23 ± 1	-

## 3 Results

**Figure 2:**
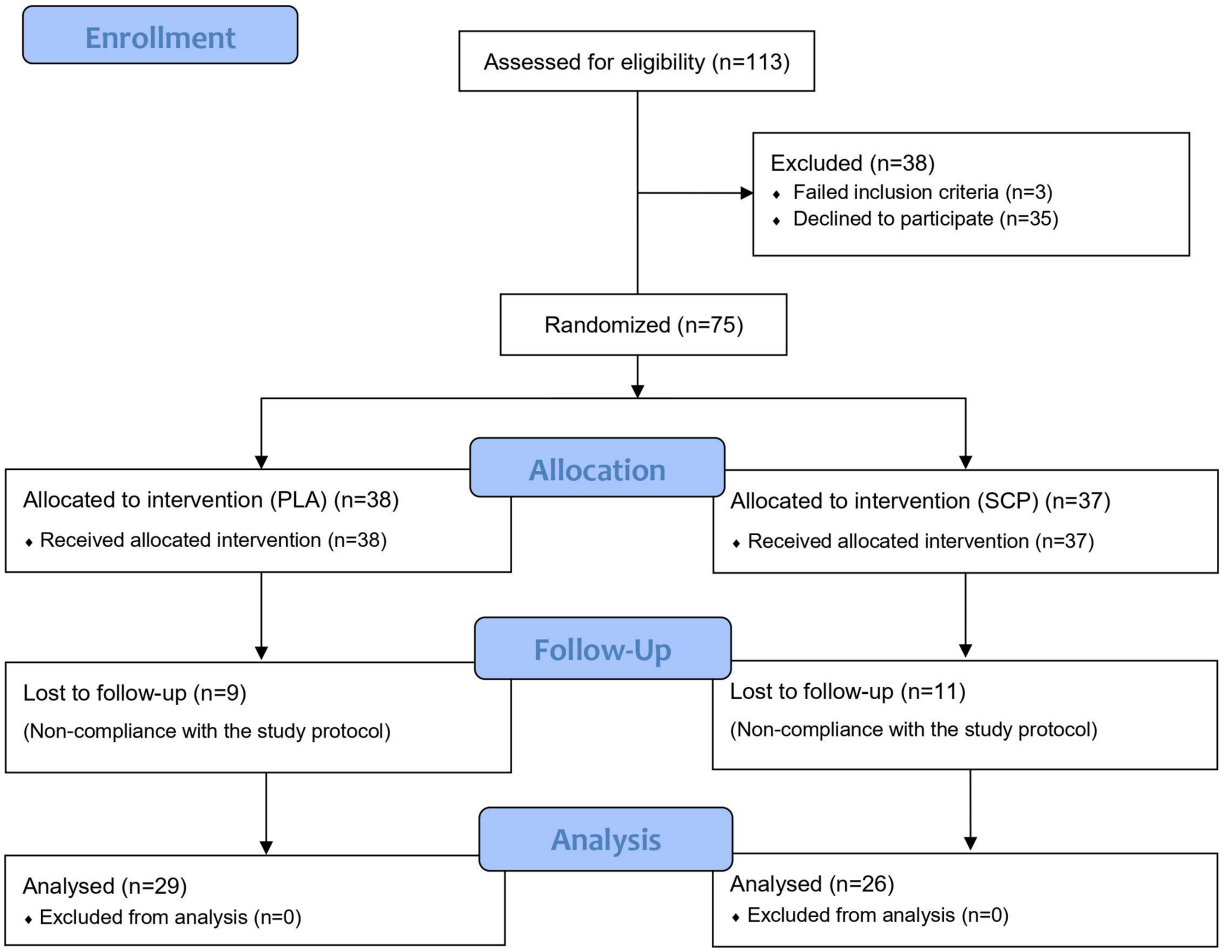
CONSORT flow chart.

After meeting the inclusion criteria and a positive medical examination, 75 participants (initially 76, but one subject declined further participation before day 1) were randomized and assigned to the SCP (*n* = 37) or PLA group (*n* = 38) (Figure 2). Participants lost to follow-up were excluded for non-compliance (missing more than 10 sessions) with the training protocol. No adverse events or side effects related to SCP or PLA supplementation were observed. 55 subjects successfully completed the study.

### 3.1 Anthropometrics and dietary intake

Regarding anthropometric parameters (Table 3), no significant differences were observed for age (*p* = 0.44), mass (*p* = 0.35), height (*p* = 0.51) and BMI (*p* = 0.7). No significant time effect was calculated for PLA [energy (*p* = 0.77), protein (*p* = 0.54), fat (*p* = 0.44), carbohydrate (*p* = 0.26)] and SCP [energy (*p* = 0.09), protein (*p* = 0.47), fat (*p* = 0.74), carbohydrate (*p* = 0.06)] in relation to dietary intake. Similarly, no differences were evident between groups in T1 [energy (*p* = 0.71), protein (*p* = 0.72), fat (*p* = 0.55), carbohydrate (*p* = 0.89)] as well as in T2 [energy (*p* = 0.08), protein (*p* = 0.09), fat (*p* = 0.08), carbohydrate (*p* = 0.52)]. In addition, no interaction effect was observed for energy (*p* = 0.24), protein (*p* = 0.32), fat (*p* = 0.36) and carbohydrate intake (*p* = 0.59).

**Table 3:** Subject characteristics and dietary intake without supplement represented as mean ± SD.

Variable	SCP (*n* = 26)		PLA (*n* = 29)		Mixed ANOVA (*p* value)
	T1	T2	T1	T2	
Age (years)	26.1 ± 5.1	-	27.2 ± 5.2	-	-
Body mass (kg)	75 ± 10	-	77.1 ± 6.3	-	-
Height (m)	1.81 ± 0.1	-	1.82 ± 0.1	-	-
BMI (kg/m^2^)	22.8 ± 2	-	23.3 ± 1.8	-	-
Energy (kcal)	2276 ± 663	2104 ± 429	2360 ± 640	2404 ± 513	0.24
Protein (g)	91 ± 31	85 ± 20	93 ± 33	98 ± 23	0.32
Fat (g)	83 ± 25	81 ± 20	89 ± 38	96 ± 30	0.36
Carbohydrate (g)	261 ± 91	234 ± 77	265 ± 58	250 ± 52	0.59

### 3.2 Blood biomarkers

Descriptive statistics for CK, LDH, MYO and hsCRP are shown in Table 2, which delineates the data used for further analyses. As some blood samples could not be obtained (entirely) from all subjects who completed the study due to malaise and nausea during the process of blood collection, the number of participants differed slightly between each marker. Individuals who failed to furnish blood at a particular time regarding one specific parameter were excluded whereas an exclusion was not obligatory for other parameters, if the amount of collected blood was sufficient for their analysis. Therefore, the following numbers of participants have been analyzed: 45 (CK), 43 (LDH), 44 (MYO) and 40 (hsCRP).

### 3.3 Area under the curve (AUC)

**Figure 3:**
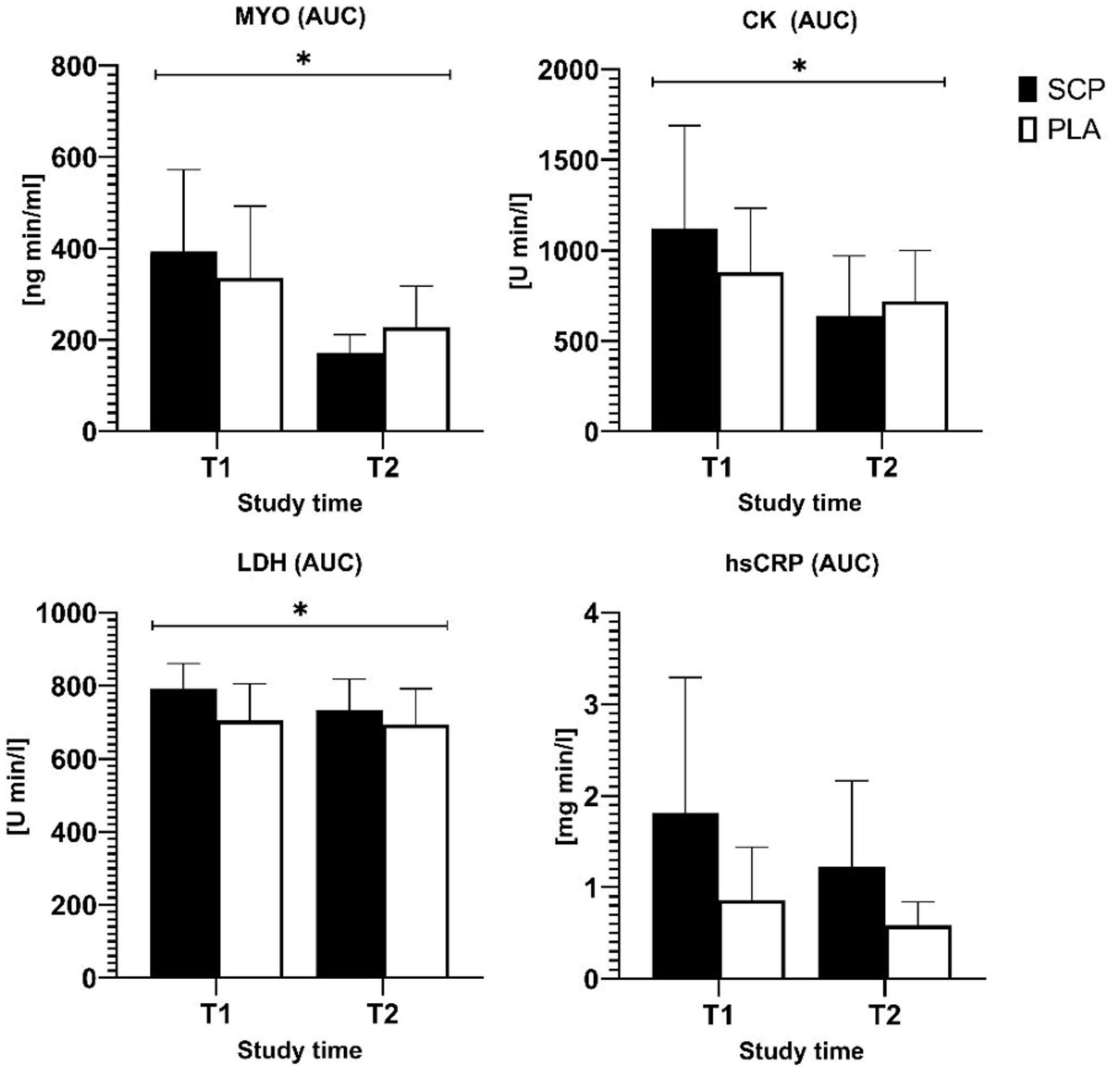
Area under the curve of MYO, CK, LDH, and hsCRP. * = significant interaction effect.

**Figure 4:**
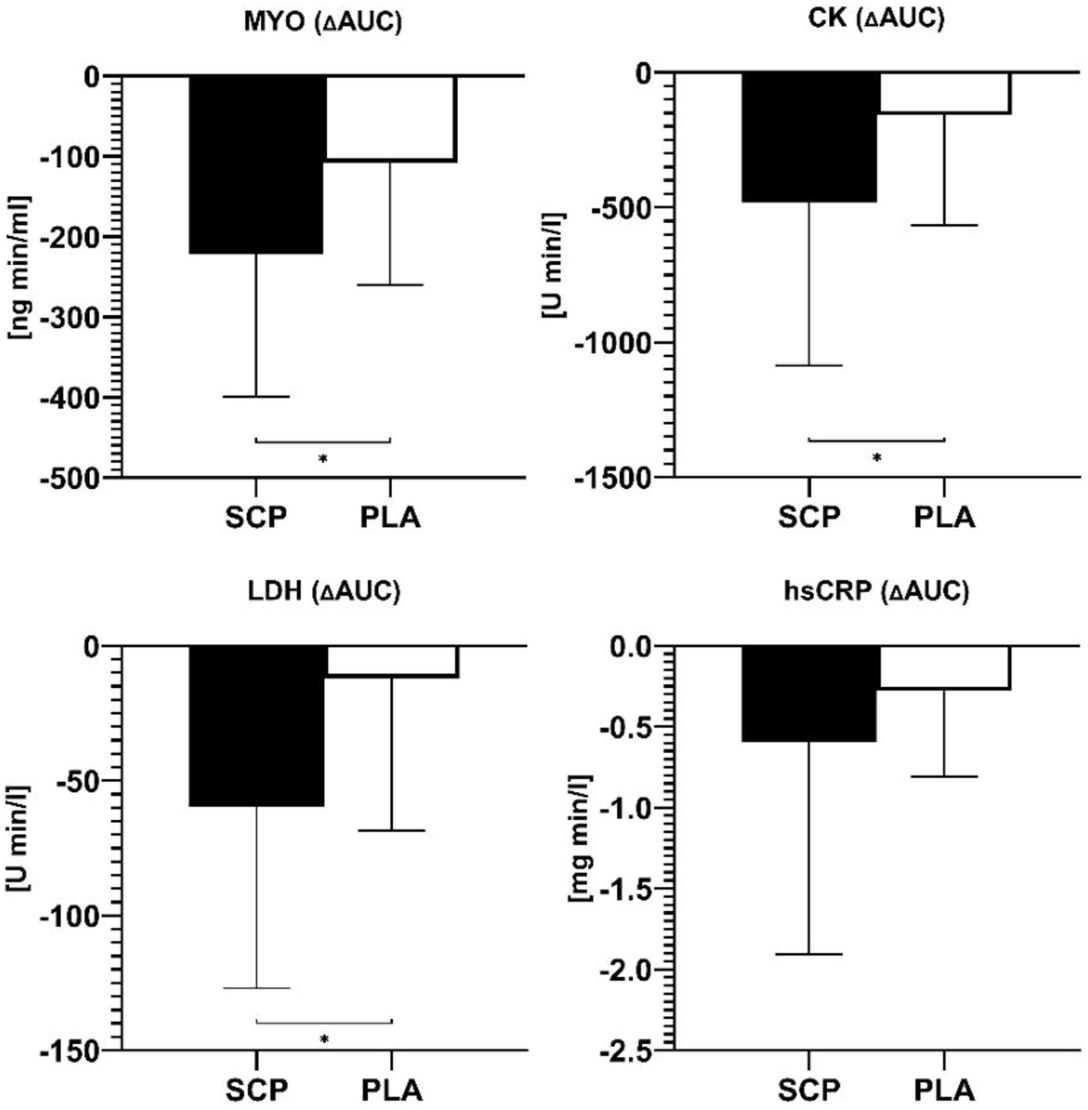
Delta AUCs of MYO, CK, LDH, and hsCRP. * = significant group difference.

The CK, MYO and hsCRP data were log-transformed due to an initial violation of normal distribution. Significant interaction effects implying a greater reduction in the SCP group were observed in the AUCs of MYO (*p* = 0.004, ηp^2^ = 0.184), CK (*p* = 0.01, ηp^2^ = 0.145), LDH (*p* = 0.016 ηp^2^ = 0.133) but not hsCRP (*p* = 0.54), as shown in Figure 3. Figures 3, 4 demonstrate the data without transformations.

### 3.4 Delta AUCs

SCP intake resulted in a significantly higher reduction, visualized by ΔAUCs, in MYO (*p* = 0.017, *d* = 0.771/ ηp^2^ = 0.129), CK (*p* = 0.039, *d* = 0.633), LDH (*p* = 0.016, *d* = 0.764) but not in hsCRP (*p* = 0.915) (Figure 4). For MYO and hsCRP, the Mann–Whitney U Test was applied as a non-parametric alternative.

## 4 Discussion

To the best of our knowledge, this is the first study to examine the longer-term impact of a daily intake of specific collagen peptides (SCP) in combination with a 12-week concurrent training (CT) program on muscle stress and recovery-related blood biomarkers. 15 g of SCP significantly decreased the area under the curve (AUC) as well as the ΔAUCs of myoglobin (MYO), creatine kinase (CK) and lactate dehydrogenase (LDH) activity, suggesting improved regenerative capacity within 48 h after muscle damage-inducing bouts of drop jumps.

The results showed better adaptation after eccentric exercise, suggesting a more pronounced repeated bout effect (RBE) after prolonged SCP ingestion. The RBE, a not yet fully understood mechanism that protects tissues involved in muscle lengthening exercise execution from subsequent damage, is thought to be due to neural adaptations, extracellular matrix remodeling, adaptations at the muscle-tendon junction, and inflammation (34).

Severe myofibrillar damage, particularly as it occurs after unaccustomed eccentric exercise bouts, usually results in significant increases in CK, LDH and MYO, which have been extensively used as indirect biomarkers to monitor the extent of acute stress as well as recovery (35). The activity of both cytosolic enzymes (CK, LDH) during a nine (10) and 33-day (13) CP supplementation period was not lower compared to a placebo after muscle-damaging exercise, which could have indicated either reduced acute muscle stress or accelerated reconstruction of damaged tissue. These two studies are the only ones to date that have examined recovery after CP intake by analyzing CK & LDH, albeit in a more short-term manner without prolonged exercise intervention. Therefore, the three-month training intervention in the present study may have improved the remodeling of the force-producing and transmitting tissues (ECM, muscle) with regular SCP administration, reducing the increase in CK & LDH. This hypothesis is supported by the results of a proteomic approach following a 12-week intake of 15 g CP and resistance training (14). The authors identified a 14-fold change in hypoxia-inducible factor alpha 1 (HIF-1α), the alpha subunit of the transcription factor HIF-1, which has shown its contribution to skeletal muscle regeneration in mice (36). HIF-1 exhibits upregulated expression in hypoxic situations, such as exercise, and has been found to play a role in collagen production in rodent tubular epithelial cells (37). Specifically, HIF-1 induces the expression of prolyl and lysyl hydroxylase, two enzymes essential for fiber formation, and promotes proper collagen biogenesis, ECM organization, stiffness, cell adhesion, and motility in human fibroblasts (38). Heat shock protein 90 (HSP90), also upregulated 14-fold in CP in (14), is an HSP chaperone group protein. HSPs are usually overexpressed when exposed to infection, heat-induced stress, hypoxia and thus heat treatment (used as a therapeutic approach), and exercise. Previous studies in mice indicated increased protein levels and markers of muscle hypertrophy/recovery (MHCneo) associated with higher HSP72 levels following downhill running (39). Data from healthy human subjects running downhill for 45 min have shown a negative correlation of ΔMYO and ΔCK along with increasing concentrations of ΔHSP90 (1 h minus baseline) 1 hour after the eccentric endurance bout (40). In addition, Heat shock factor 1 (HSF1), a major transcription factor of HSPs, was also upregulated in CP influenced by HIF-1α in (14), indicating a close interdependence of these proteins (41). It appears that HSPs are involved in protecting cells from damage (42). Still, caution is also warranted when persistently elevated levels are observed, as they indicate chronic inflammation (43). Finally, five and four-fold changes in MAPK and PI3K-Akt pathways have been observed after CP ingestion (14), which are important initiators of signaling cascades leading to protein synthesis. There is little evidence from *in-vitro* experiments that PI3K-Akt & MAPK may also trigger collagen type I gene expression via TGF-β2 activation (16, 44, 45). TGF-β cytokines generally induce intracellular signaling via specific Smad proteins (Smad2, 3 forming Smad 4 etc.) (46), the latter being responsible for the expression of COL1A2 and COL3A1 (47) which give rise to collagen types I & III, both of which are most abundant in intramuscular connective tissue (48, 49). Given that Smad2, 4 and COL1A1 & COL3A1 mRNA were significantly upregulated 27 (COL1A1) and 30 days after two muscle-damaging bouts of eccentric contractions separated by 27 days (50), the remodeling of muscle ECM, particularly force-bearing proteins such as COL I & III, represents a longer-term process (51). This functional recovery and adaptation process appears to be enhanced by CP supplementation induced by the mechanisms described above.

Based on the CK, LDH & MYO results, the findings suggest increased regenerative capacity throughout ongoing myocellular remodeling. While LDH typically peaks within hours, MYO concentrations and CK activity are highest on the first to third day or, in some cases, even on the fourth day after strenuous exercise, depending on the type of load, muscles used, and individual training history (52–57). Each biomarker leaves its original intracellular location due to cell damage-induced permeability and subsequently enters the bloodstream, where it can be measured in serum/plasma in chronological order (LDH > MYO > CK) (35, 58). In the present study, prolonged SCP intake was shown to ameliorate several cell damage markers and have a beneficial effect on muscle stress response, early-phase muscle recovery, and enhanced RBE. Whether adaptions occurred in surrounding structures such as intramuscular connective tissue and/or muscle fibers *per se* remains unknown.

Regarding inflammatory parameters, the acute-phase protein C-reactive protein (CRP) is a commonly used marker of local and systemic inflammation. In this trial, there was no significant difference between both groups in terms of hsCRP. Considering the measurement time points (baseline and after 24 h), significant CRP changes were already detected after a marathon race (59), a one-hour strenuous endurance run (52), a 90-min eccentric exercise protocol (60) and 100 drop jumps (61). SCP intake had no apparent significant effect on post-exercise inflammatory status in this study; among other factors, the high CV of 12.5% for hsCRP might be responsible for this.

The choice of concurrent training (CT) as the type of training intervention in the present study was based on the following considerations. In general, CT combines the benefits of both RT and ET, at least in untrained to recreational athletes. CT improves physical fitness, does not impair maximal strength and muscle hypertrophy, and is thus consistent with public health guidelines (62). In contrast, CT limits explosive strength gains compared to resistance-only training, offset in the current study by performing RT before ET (63). In agreement with (64), moderate to high ET intensity was prescribed to benefit cardiorespiratory fitness and muscle strength (64). As a recent systematic review mentioned, protein intake (whey, casein, soy, milk) after an acute CT bout increases myofibrillar protein synthesis to a similar extent as after RT (65). Moreover, phosphorylation of the signaling proteins Akt–mTOR-S6K increased significantly immediately after CT and protein intake compared with placebo, indicating enhanced translation of myofibrillar mRNAs (66). CT plus protein also resulted in an immediate post-exercise increase in specific micro RNAs, suggesting a more myogenic rather than oxidative adaptation, and an effect on longer-term CT responses (65, 67). Whether SCP supplementation in combination with CT also leads to the above results remains unknown but may be of interest for future studies.

### 4.1 Limitations

The present study also has some limitations. Selecting an exercise that also stressed upper limb muscles (e.g., elbow flexors) would have led to more insightful information about the recovery-related setting of blood markers, as upper and lower body muscles have been shown to elicit a slightly different response to MYO & CK (57, 68). In addition, it has recently been suggested that a 30 g dose of milk protein taken immediately after endurance training efficiently repairs and resynthesizes body proteins (65, 69). Whether the same amount of SCP supplementation over a longer period would result in improved recovery of tissues subjected to exercise-induced muscle damage requires future investigation. To date, there is no clinical evidence or recommendations for effective CP dosing to optimize regenerative capacity. In addition, the effects of SCP supplemented in the present study cannot be generalized to other CP products.

### 4.2 Conclusion

In conclusion, 12 weeks of daily supplementation of 15 g of specific collagen peptides (SCP) in combination with a concurrent training intervention resulted in reduced acute muscle stress response and improvements in early phase recovery, as represented by significantly lower levels of myoglobin (MYO), creatine kinase (CK), and lactate dehydrogenase (LDH) after a second bout of muscle damage-inducing exercise. As this also indicates an enhanced repeated bout effect (RBE), SCPs, together with training, may accelerate repair and reduce cellular damage to the force-producing and transmitting tissues in the first 48 h after eccentric exercise. Future studies should therefore investigate the underlying molecular pathways of myocellular and ECM adaptation after intense muscle-damaging exercises. This may reveal specific target mechanisms affected by SCP intake that could be of significant practical importance in reducing rehabilitation and recovery time and improving RBE.

## Data availability statement

The raw data supporting the conclusions of this article will be made available by the authors, without undue reservation.

## Ethics statement

The studies involving humans were approved by Ethics Committee of the University of Vienna (reference no. 00765). The studies were conducted in accordance with the local legislation and institutional requirements. The participants provided their written informed consent to participate in this study.

## Author contributions

KB: Writing – original draft, Writing – review & editing. SS: Writing – review & editing. LB: Writing – review & editing. SO: Writing – review & editing. AB: Writing – review & editing. DK: Writing – original draft, Writing – review & editing.

## Footnotes

^1^ https://www.randomizer.org
